# Amniotic mesenchymal stem cells display neurovascular tropism and aid in the recovery of injured peripheral nerves

**DOI:** 10.1111/jcmm.12249

**Published:** 2014-04-08

**Authors:** YongNan Li, Longzhe Guo, Hyun Sook Ahn, Moo Hyun Kim, Sung-Whan Kim

**Affiliations:** a Department of Neurology, The Fourth Affiliated Hospital, Harbin Medical UniversityHarbin, Heilongjiang, China; b Department of Cardiology, College of Medicine, Dong-A UniversityBusan, Korea; c Regional Clinical Trial Center, Dong-A University HospitalBusan, Korea; d Department of Anatomy and Cell Biology and Mitochondria Hub Regulation Center, College of Medicine, Dong-A UniversityBusan, Korea; e Department of Obstetrics and Gynecology, Ilsin Christian HospitalBusan, Korea; f Innovative Cell & Gene Therapy Center, International St.Mary's HospitalIncheon, Korea

**Keywords:** amniotic stem cells, angiogenesis, cell therapy, engraftment, peripheral nerve

## Abstract

Recently, we reported that human amniotic membrane-derived mesenchymal stem cells (AMMs) possess great angiogenic potential. In this study, we determined whether local injection of AMMs ameliorates peripheral neuropathy. AMMs were transplanted into injured sciatic nerves. AMM injection promoted significant recovery of motor nerve conduction velocity and voltage amplitude compared to human adipose-derived mesenchymal stem cells. AMM implantation also augmented blood perfusion and increased intraneural vascularity. Whole-mount fluorescent imaging analysis demonstrated that AMMs exhibited higher engraftment and endothelial incorporation abilities in the sciatic nerve. In addition, the higher expression of pro-angiogenic factors was detected in AMMs injected into the peripheral nerve. Therefore, these data provide novel therapeutic and mechanistic insights into stem cell biology, and AMM transplantation may represent an alternative therapeutic option for treating peripheral neuropathy.

## Introduction

Peripheral nerve injury is a devastating complication that can lead to complete functional loss or permanent impairment. Despite significant advances in the repair of peripheral nerves, the functional recovery of these nerves never returns to the pre-injury state. Therefore, there have been many experimental trials to repair injured nerves [1]. Recently, alternative materials embedded with regenerative cells were evaluated [2,3].

A novel therapeutic method using cells has been proposed for regenerating damaged nerves [4]. Schwann cells or stem cells derived from various tissues were transplanted and contributed to peripheral nerve repair. Secreted neurotrophic factors that cause differentiation towards Schwann(–like) cells stimulate myelination or the growth of nerve sprouts [5]. However, there are limitations for the use Schwann cells because of the difficulties of collection and expansion *in vitro*. By contrast, mesenchymal stem cells (MSCs) are easily obtained and can be expanded *in vitro*. MSCs secrete VEGF, FGF and HGF, and MSCs derived from adipose tissue stimulate nerve growth in the ischaemic myocardium and promote peripheral nerve repair [6]. In addition, amniotic fluid-derived MSCs promote peripheral nerve regeneration by secreting neurotrophic factors [7].

Stem cell-based therapies have appeared as a promising strategy for injured tissue regeneration [8]. Recently, we reported the higher angiogenic and chemotactic properties of amniotic membrane-derived mesenchymal stem cells (AMMs) compared to adipose tissue-derived MSCs (ADMs) [9–11]. However, the therapeutic potential of AMMs in peripheral nerve injury has not been fully elucidated. In this study, we determined whether neuropathy can be ameliorated by the local transplantation of AMMs.

## Materials and methods

### Cell culture

Amniotic membrane-derived mesenchymal stem cells and human ADMs were purchased from Thermo Scientific Inc. (Rockford, IL, USA). The manufacturer provided the multi-lineage differentiation potential and cell characterization data for these MSCs. The AMMs and ADMs were cultured in low-glucose DMEM (Gibco, Grand Island, NY, USA) supplemented with 10% fetal bovine serum, 100 U/ml penicillin and 100 μg/ml streptomycin (Gibco). The Institutional Review Boards of the Dong-A University approved all protocols involving human samples and the study conformed to the principles established in the Declaration of Helsinki.

### Real-time polymerase chain reaction (PCR) analysis

Quantitative real-time (qRT)-PCR assays were performed as previously described [12]. In brief, the total RNA was isolated from nerve tissues by using RNA-stat (Iso-Tex Diagnostics, Friendswood, TX, USA) according to the manufacturer's instructions. The extracted RNA was subsequently reverse transcribed by using Taqman Reverse Transcription Reagents (Applied Biosystems, Foster City, CA, USA). The synthesized cDNA was subjected to qRT-PCR by using primers. The RNA levels were quantitatively assessed by using an ABI PRISM 7000 Sequence Detection System (Applied Biosystems). The relative mRNA expression was normalized to Glyceraldehyde-3-phosphate dehydrogenase (GAPDH) expression and was calculated as described previously [12]. The primers used for qRT-PCR were mouse Ang-1 (Mm00456503_m1), Fgf-1 (Mm01258325_m1), Fgf-2 (Mm01285715_m1), Igf-1 (Mm00439560_m1), Vegf-a (Mm01204733_m1), and GAPDH (Mm99999915_g1). We purchased the primer/probe sets from Applied Biosystems.

### Transplantation of cells in the sciatic nerve injury model

The Dong-A University Institutional Animal Care and Use Committee approved the experimental protocols, and all procedures were performed in accordance with the Guide for the Care and Use of Laboratory Animals published by the U.S. National Institutes of Health (NIH Publication No. 85-23, revised 1996). Male NOD/severe combined immunodeficiency (scid) mice (NOD.CB17-Prkdc^scid^/J strain; The Jackson Laboratory, Bar Harbor, ME, USA) ageing 8–9 weeks old and weighing 19–23 g were used. The mice were anaesthetized with isoflurane (induction: 450 ml air, 4.5% isoflurane, maintenance: 200 ml air, 2.0% isoflurane; Baxter International, Inc., Deerfield, IL, USA), and the depth of anaesthesia was monitored by respiratory rate and the lack of withdrawal reflex upon toe pinching as previously described [11]. The sciatic nerve was exposed and crushed at the mid-thigh level for 15 sec. by using a haemostat as previously described [13]. The mice were injected with 1 × 10^6^ 1,1–dioctadecyl-3,3,3′,3′-tetra-methylindocarbocyanine (DiI) dye-labelled AMMs and ADMs in 100 μl PBS or the same volume of PBS intramuscularly in the muscles along the sciatic nerve at four sites.

### Laser Doppler perfusion imaging (LDPI)

Blood perfusion was measured at 4 weeks after the operation as previously described [14]. Briefly, the mice were anaesthetized and placed on a heating blanket to maintain a constant temperature. The nerves were exposed by using blunt dissection and scalpel incision. The blood flow in the sciatic nerve was examined by using LDPI (Moor Instrument, Wilmington, Delaware).

### Neurophysiological measurement

A nerve conduction assay was performed with a TECA TD-10 (Oxford Instruments, Pleasantville, NY, USA). The motor nerve parameters and compound muscle action potentials were measured as previously described [15]. The motor conduction velocity (MCV) was calculated by dividing the distance between the stimulating electrodes by the average latency difference [15].

### Behavioural testing

Behavioural testing was performed as previously described [15]. Briefly, mice were trained six times for 2 weeks before surgery. After sciatic nerve injury, mice were placed on a rotarod treadmill and the maximum duration was calculated. The speed was from 4 to 40 rpm. The test was ended if mice gripped the device or fell off.

### Fluorescent imaging of vasculature in sciatic nerve

Fluorescent imaging of the vasculature was performed with modification as described previously [14]. Briefly, after anaesthesia, the aortas of the mice was catheterized and perfused with BS-1 lectin conjugated to FITC (Vector Laboratories, Burlingame, CA, USA). To facilitate *in situ* staining, the inferior vena was ligated. Fifteen minutes later, the mice were killed and the sciatic nerves were collected and fixed with 4% paraformaldehyde. The nerve tissues were whole mounted for longitudinal observation.

### Histological analysis

The sciatic nerves were harvested, fixed in 4% paraformaldehyde for 4 hrs, and incubated overnight in a 15% sucrose solution. The tissues were embedded in OCT compound (Sakura Finetek USA, Torrance, CA, USA), snap frozen in liquid nitrogen, and cross sectioned at a thickness of 10 μm. For capillary density measurement, six frozen sections from each group of sciatic nerve tissues were stained with biotinylated isolectin B4 (ILB4, 1:250; Vector Laboratory Inc.) primary antibodies followed by streptavidin Alexa Fluor 488 (1:400; Invitrogen, Carlsbad, CA, USA) secondary antibodies. Five fields from six tissue sections were randomly selected, and the number of capillaries was counted in each field. Photographs were taken by using confocal microscopy.

### Statistical analysis

Statistical analyses were performed with Student's *t*-test for comparisons of the two groups, and anova with Bonferroni's multiple comparison tests was also performed with SPSS v12.0 (SPSS Inc., Chicago, IL, USA). All data were presented as the mean ± SD. A value of *P* < 0.05 was considered statistically significant.

## Results

### Local injection of AMMs improve nerve function

**Figure 1:**
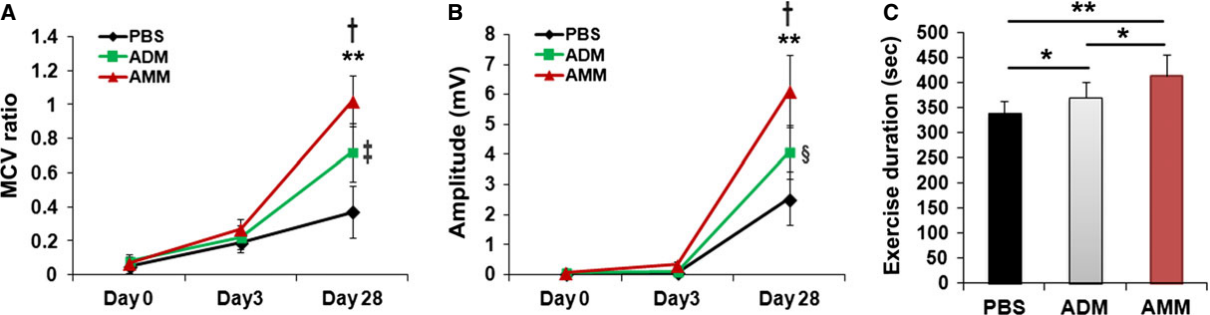
Amniotic membrane-derived mesenchymal stem cells (AMMs) transplantation promotes functional and physiological recovery in damaged sciatic nerves. (A) Motor nerve conduction velocity (m/sec.) and (B) nerve voltage amplitude (mV) were examined for 4 weeks after nerve injury. **P < 0.05 AMMs *versus* PBS, ^†^*P* < 0.05 AMMs *versus* adipose-derived mesenchymal stem cells (ADM)s, ^‡^*P* < 0.01 ADMs *versus* PBS, ^§^*P* < 0.01 ADMs *versus* PBS. *n* = 9 per group. (C) Exercise duration was assessed at 4 weeks after nerve injury. **P < 0.01, *P < 0.05; *n* = 6 per group.

To test the therapeutic effects of local injection of AMMs after nerve injury, we measured the MCV for 4 weeks after treatment. There were no significant differences on day 3 after the injury in all treated groups. However, on day 28, the mice treated with AMMs exhibited significantly improved recovery compared to the mice treated with the ADMs and the PBS control groups (1.02 ± 0.14 *versus* 0.72 ± 0.17, *P* = 0.021 and 0.37 ± 0.14, *P* = 0.003, respectively; Fig.1A).

Nerve functional recovery was also examined by using nerve voltage amplitude. After 4 weeks, the mice that were transplanted with the AMMs showed significantly higher amplitude compared to the ADMs and PBS control groups (6.12 ± 1.2 *versus* 4.11 ± 0.8, *P* = 0.024 and 2.55 ± 0.0.9, *P* = 0.002, respectively; Fig.1B).

Next, to examine the recovery of motor coordination, behavioural testing was performed with rotarod treadmill. The mice treated with AMMs highly recovered their ability to conduct the rotarod task compare to the ADMs and PBS control groups (412 ± 43 *versus* 371 ± 29, *P* = 0.042 and 338 ± 26, *P* = 0.001, respectively; Fig.1C).

### AMMs increase blood perfusion and vascularization of nerves

**Figure 2:**
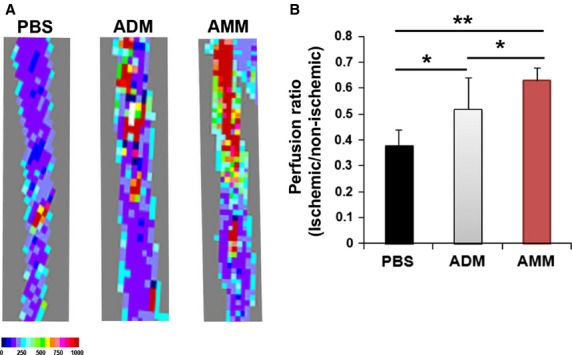
Amniotic membrane-derived mesenchymal stem cells (AMMs) transplantation augments blood perfusion. (A) Representative pictures of blood perfusion in the sciatic nerve. (B) Quantitative analysis by using laser Doppler perfusion imaging. AMMs significantly improved blood perfusion compared to adipose-derived mesenchymal stem cells and PBS. **P < 0.01, *P < 0.05; *n* = 9 per group.

To determine whether the local injection of AMMs promotes blood circulation to the nerve, we examined the blood flow of the sciatic nerves by using LDPI. The blood flow of the nerves was 1.6- and 1.2-fold higher in the AMM-injected nerves than the ADM- (*P* = 0.03) or PBS-injected (*P* < 0.001) nerves (Fig.2A and B).

**Figure 3:**
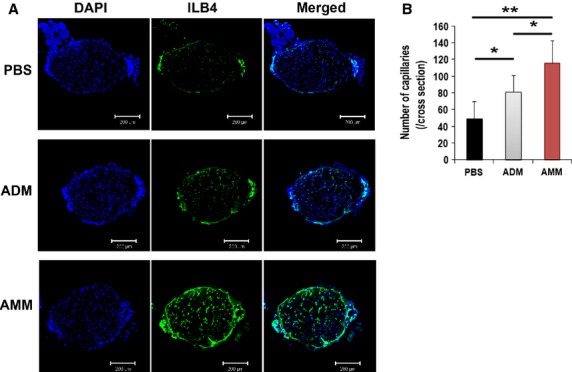
Amniotic membrane-derived mesenchymal stem cells (AMMs) transplantation restores vascularity. (A) Representative images of cross-sectioned sciatic nerves. (B) Quantitative evaluation of capillary density. AMMs significantly increased the vascularity compared to adipose-derived mesenchymal stem cells and PBS. **P < 0.01, *P < 0.05; *n* = 7 per group.

Next, to identify changes in the blood vessels in the sciatic nerve, we quantified the capillaries. We harvested nerves 4 weeks after cell injection and visualized the capillaries of the nerves by using FITC-conjugated isolectin (ILB4). Cross sections of the nerves revealed that the capillaries were 2.3- and 1.4-times higher in the AMM-transplanted nerves (*P* = 0.0005, *P* = 0.019, respectively) than the PBS-injected or ADM-injected nerves (Fig.3A and B).

### AMMs highly engraft to nerves and vasa nervorum

**Figure 4:**
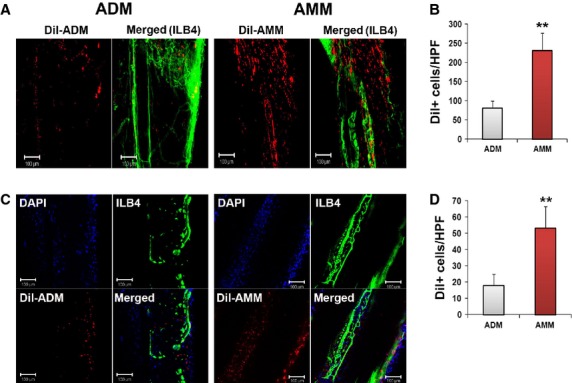
Amniotic membrane-derived mesenchymal stem cells exhibit high engraftment potential in nerves. (A) Representative images of longitudinal whole-mount nerves show engrafted transplanted stem cells in the perineurium area 4 weeks after cell transplantation. (B) Quantitative evaluation of engrafted transplanted stem cells in the perineurium area. **P < 0.01, *P < 0.05; *n* = 7 per group. (C) Representative images of longitudinal whole-mount nerves show engrafted transplanted stem cells in the vasa nervorum 4 weeks after cell transplantation. (D) Quantitative evaluation of engrafted transplanted stem cells in the vasa nervorum. **P < 0.01, *P < 0.05; *n* = 7 per group.

The engraftment or survival potentials of the AMMs were investigated in an injured sciatic nerve. One million Dil-labelled cells of each type were directly transplanted into the muscle along the injured sciatic nerve. Four weeks after cell transplantation, the sciatic nerve tissues were collected and analysed by immunohistochemistry. The immunohistochemistry results demonstrated that AMMs exhibited a significantly higher engraftment potential (237.0 ± 45.5 *versus* 81.3 ± 18.0, *P* = 0.002) in the perineurium (Fig.4A and B) and (53.3 ± 13.5 *versus* 13.5 ± 7.1, *P* = 0.008) vasa nervorum area (Fig.4C and D).

**Figure 5:**
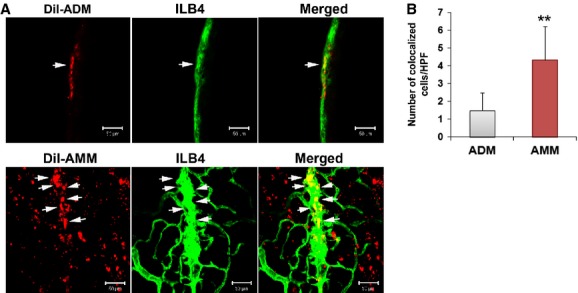
Amniotic membrane-derived mesenchymal stem cells exhibit high incorporation potential with vasculature in the nerve. (A) Representative images of longitudinal whole-mount nerves show that transplanted stem cells colocalize with the vascular endothelium in the sciatic nerve 4 weeks after cell transplantation. (B) Quantitative evaluation of transplanted stem cells that colocalize with the vascular endothelium in the sciatic nerve. **P < 0.01, *P < 0.05; *n* = 7 per group.

The majority of transplanted cells were localized in the epineurium or perivascular areas. However, a low number of transplanted cells were colocalized with the vasa nervorum. Quantification analysis revealed that a significantly higher number of AMMs incorporated with the vasa nervorum compared to the ADMs (*P* = 0.005; Fig.5A and B).

### AMMs increase the level of angiogenic factor in the sciatic nerve

**Figure 6:**
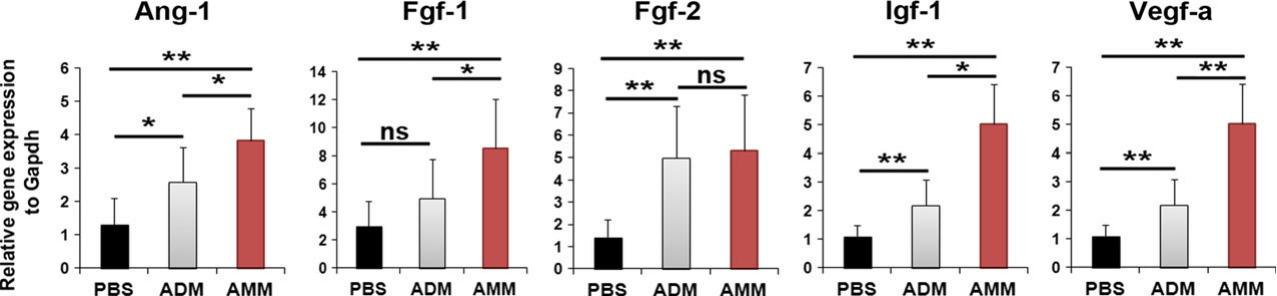
Amniotic membrane-derived mesenchymal stem cells (AMMs) increase the level of angiogenic factor in the sciatic nerve. Increased expression of angiogenic factors was detected in sciatic nerve tissues injected with AMMs. **P < 0.01, *P < 0.05; *n* = 7 per group.

To elucidate the mechanisms underlying the improvement in nerve function following AMM injection, we measured the expression of pro-angiogenic genes in the sciatic nerves 2 weeks after cell transplantation. Quantitative RT-PCR results revealed that the expression levels of pro-angiogenic genes, such as Ang-1, Fgf-1, Igf-1 and Vegf-a were significantly higher in the AMM-transplanted nerve than the ADM- and PBS-transplanted nerves (Fig.6). However, the mRNA level of Fgf-2 was not significantly different between the AMMs and ADMs.

## Discussion

To the best of our knowledge, we are the first to demonstrate that AMMs display neurovascular tropism and that the local injection of AMMs improves neuropathy. We report several important findings in this study. First, the local transplantation of AMMs restored the NCVs of nerves. Second, AMM injection induced the revascularization of nerves. Third, the injected AMMs highly engrafted in the sciatic nerve and incorporated with the vasa nervorum. Fourth, the expression of angiogenic factors in the nerves was increased by AMM implantation.

Recently, we investigated AMMs to characterize their therapeutic effects for allogeneic cell therapy. A large amount of AMMs can be easily obtained and isolated from discarded placentas, and these AMMs have high proliferation and transdifferentiation capacities [9,11]; and neurogenesis requires angiogenesis about 7 days after damage, indicating that angiogenesis plays a critical role in the neurogenesis of injured nerves [15,16]. Therefore, we hypothesized that the robust angiogenic and chemotactic activities of the AMMs may ameliorate neuropathy. Here, we demonstrated that treatment with AMMs improved the functional recovery of nerves. These results might attribute to the migration of AMMs into nerve, following that they secreted angiogenic factors such as VEGF inside and outside of the nerve. Consistent with these speculations, previous reports also demonstrated that administration of VEGF reduces infarct size of brain [17] and improves neurological outcome [18]. The establishment of a vascular niche might stimulate the proliferation or differentiation of neuronal precursors [19]. For the understanding of more precise therapeutic mechanism, however, further studies focused on neurogenesis after stem cell transplantation might be necessary.

To investigate the therapeutic mechanism of cell transplantation in nerve injury, we examined the number of blood vessels and the fate of the injected cells. In this study, the increased capillary density and some of the transplanted AMMs and ADMs expressed an endothelial marker (ILB4). The majority of transplanted MSCs did not incorporate with the vasa nervorum, indicating that the main therapeutic mechanism is angiogenesis. Additional data supporting this hypothesis is the increase in multiple angiogenic factors, such as Ang-1, Fgf-1, Igf-1 and Vegf-a, which were detected in the nerve after cell transplantation. These up-regulated angiogenic factors may display neurotrophic effects and reverse neuropathy. Taken together, these results suggest that the vasa nervorum plays a critical role in neuropathy and that vascular defects are associated with many neurological disorders. In fact, there are reports that the administration of angiogenic factors, such as VEGF or Sonic hedgehog (Shh) improved neuropathy [14,20].

Because of the low cell survival or engraftment characteristics of the transplanted stem or progenitor cells [21], marginal therapeutic effects have been a major obstacle in cell therapy. Recently, we reported that AMMs highly express Akt and exhibit elevated levels of Akt phosphorylation [11], which exerts anti-apoptotic effects through downstream targets and suppresses cell death. Specifically, a growing body of evidence suggests that Akt mediates neuronal survival or protection [22–24]. In addition, AMMs have a high adhesion property, which plays a key role in engraftment [10]. Consistent with these data, the AMMs showed high engraftment properties in the injured nerve, indicating a benefit for treating peripheral nerve disease.

In this study, we observed an intriguing characteristic of the transplanted MSCs, homing potential. Transplanted MSCs preferentially localize to the perineurium and are specifically positioned near the vasa nervorum. To the best of our knowledge, this tropism of MSCs towards injured peripheral nerves has not been previously reported. In addition, the transplanted AMMs exhibited higher engraftment in the nerve and colocalization potential with the vasa nervorum compared to the ADMs. Consistent with theses data, we recently reported that AMMs highly express chemokine genes, such as IL-8 and GCP-2 and chemokine receptors, CCR2, CCR3 and CCR5 [10]. Amniotic membrane-derived mesenchymal stem cells also display enhanced cell migration, engraftment and endothelial transdifferentiation properties. Therefore, we hypothesized that these chemotactic properties of AMMs may mediate neovascular tropism and neurotropic effects. However, additional studies regarding the molecular mechanisms of tropism are required.

In conclusion, we demonstrated that AMM transplantation improved the functional outcome in sciatic nerve injury. To the best of our knowledge, this is the first report linking the therapeutic potency of AMMs and showing better improvement than ADMs in the treatment of injured peripheral nerves. Therefore, we postulate that the transplantation of AMMs may be a promising alternative therapeutic option to treat peripheral nerve diseases.
